# Characterization and prediction of acute and sustained response to psychedelic psilocybin in a mindfulness group retreat

**DOI:** 10.1038/s41598-019-50612-3

**Published:** 2019-10-24

**Authors:** Lukasz Smigielski, Michael Kometer, Milan Scheidegger, Rainer Krähenmann, Theo Huber, Franz X. Vollenweider

**Affiliations:** Neuropsychopharmacology and Brain Imaging, Department of Psychiatry, Psychotherapy and Psychosomatics, Psychiatric University Hospital Zurich, University of Zurich, Lenggstrasse 31, CH-8032 Zurich, Switzerland

**Keywords:** Human behaviour, Psychiatric disorders

## Abstract

Meditation and psychedelics have played key roles in humankind’s search for self-transcendence and personal change. However, neither their possible synergistic effects, nor related state and trait predictors have been experimentally studied. To elucidate these issues, we administered double-blind the model psychedelic drug psilocybin (315 μg/kg PO) or placebo to meditators (*n* = 39) during a 5-day mindfulness group retreat. Psilocybin increased meditation depth and incidence of positively experienced self-dissolution along the perception-hallucination continuum, without concomitant anxiety. Openness, optimism, and emotional reappraisal were predictors of the acute response. Compared with placebo, psilocybin enhanced post-intervention mindfulness and produced larger positive changes in psychosocial functioning at a 4-month follow-up, which were corroborated by external ratings, and associated with magnitude of acute self-dissolution experience. Meditation seems to enhance psilocybin’s positive effects while counteracting possible dysphoric responses. These findings highlight the interactions between non-pharmacological and pharmacological factors, and the role of emotion/attention regulation in shaping the experiential quality of psychedelic states, as well as the experience of selflessness as a modulator of behavior and attitudes. A better comprehension of mechanisms underlying most beneficial psychedelic experiences may guide therapeutic interventions across numerous mental conditions in the form of psychedelic-assisted applications.

## Introduction

A burgeoning interest in the phenomenology and neurobiology of psychedelic drugs has grown in recent years, motivated by rising evidence of their therapeutic potential for some psychiatric disorders^1,2^. For example, psilocybin reduced symptoms of therapy-resistant depression^3^ and depression and anxiety in terminal cancer patients^4^ after administration of just one or two doses. Psilocybin and other classic psychedelics are partial serotonin-2A-receptor agonists^5^ that may produce transient, profound changes in self-consciousness, experienced as a dissolution of the ordinary sense of self and a breakdown of the perceived boundaries between the self and the world^6^. Empirical research has repeatedly reported that, in a supportive setting at medium-to-high doses (20–30 mg PO), psilocybin can trigger alterations of self-consciousness in association with feelings of bliss, unity, and insightfulness^7–10^. Such experiences have been referred to as at least partially overlapping occurrences of self/ego dissolution^11,12^, self-loss^13^, states of selflessness^14–16^, mystical-type experiences^17,18^, or non-dual awareness^19^. Although the experiential quality of psilocybin-induced alterations resemble the states of self-transcendence reported by various traditions of Eastern descent^20,21^ and can occasionally occur during deep meditative practices^22,23^, there are no experimental data that support this assumption.

The intensity and form of psychedelic experience, indexed as mystical-type experience or self-dissolution effect, seem to mediate beneficial therapeutic outcomes^24–26^. Similarly, many meditation practices aim to reduce self-referential processing^27,28^ and achieve a temporary dissolution of self-boundaries^15^, which may lead to an enduring reduction of self-focus or ego-centricity^14,29,30^. Notably, meditation was found to alleviate symptoms of depression, anxiety, and stress and to promote lasting benevolent emotions and prosocial behavior^30^ in clinical and non-clinical populations^31–33^. Despite similarities between meditation- and psychedelic-induced temporary alterations in the sense of self, neither the phenomenological similarities and differences of such multifaceted experiences nor the impact of additional putative modulatory factors have yet been systematically investigated in a prospective study. The phenomenological differences between meditation- and psychedelic-induced self-dissolution may result in different long-term changes in psychosocial functions.

Many traditional meditative practices, and in particular mindfulness-based meditation such as Vipassana, Zen, and their secular forms^34^, aim at maintaining attention on the present moment with a non-judgmental, accepting stance toward the enfolding experiences^15,35,36^. This involves directing of attention, body awareness, and emotion regulation strategies to achieve an increased cognitive and emotional flexibility^35–37^ This, in turn, is thought to reduce self-centered psychological functioning^15,30^, which may culminate in states of selflessness^15,38^. Meditation experiences arise along a spectrum of meditation depth^39^ and are shaped by other factors, such as previous meditation experience, dispositional mindfulness, openness, and absorption^40,41^. However, profound states of selflessness during meditation occur rarely and mostly in long-term meditators^22^. In contrast, psilocybin can induce a state of self-dissolution at a relatively high rate (up to 60%), particularly at higher doses^42,43^, and along a perception-hallucination continuum with increasing emotional arousal.

Although the content and intensity of psychedelic experiences depend most critically on dosage^44^, the same dose can induce a pleasurable state of self-dissolution or, under certain circumstances, a more distressing response associated with thought disturbances, fear of losing control, anxiety, or panic^45^. The reactions to psychedelics and their subsequent interpretations are commonly shaped by the “set” (i.e., the subject’s expectation, personality traits, and current mood state) and “setting” (i.e., the physical and social environment in which the drug is taken)^46–49^. Considering the role of set and setting in study designs resulted in more positive subjective effects and fewer dysphoric reactions^9,50^ than in earlier experimental work that ignored these issues^51,52^. Thus, a better understanding of non-pharmacological variables for the outcome of the psychedelic experience seems crucial, in consideration of their suggested therapeutic potential.

Taken together, both meditation and psilocybin can promote a spectrum of altered states of consciousness that are modulated by additional dispositional (trait) and state factors. In this prospective, matched-group study, we quantified the effects of meditation alone and meditation combined with psilocybin in expert meditators participating in a well-structured 5-day mindfulness-based meditation retreat. First, we explored whether state mindfulness and meditation depth increased across the retreat and whether psilocybin modulated these outcomes, in addition to direct pre-post trait-like mindfulness. Second, we compared the spectrum and extent of alterations of consciousness, indexed by the Altered States of Consciousness Rating Scale (5D-ASC), and mystical-type of experience, indexed by the Mysticism Scale (M-scale). Furthermore, we conducted a multiple regression analysis to explore whether a number of trait and state factors contributed to inter-individual differences in self-dissolution as measured by the 5D-ASC and M-Scale instruments. Specifically, we hypothesized that particular state and trait variables would influence self-dissolution, i.e., openness, extraversion, neuroticism^53,54^, absorption^41,44^, optimism about life^53^, pre-experience with altered states^53^, mood and emotion regulation strategies^36,44,53^, trait and state mindfulness, and meditation depth the day before drug intake^41^. Additionally, we explored the possibility that the high cognitive and emotion regulation capacity of meditation experts^36,55–58^ would buffer possible distressing aspects of psychedelic experiences, such as negative emotions, anxiety, disorientation, and fear of loss of control and self-identity (colloquially known as a “bad trip”)^59^, which can arise with high doses of psilocybin. Because both the extent of psilocybin-induced alterations of self-consciousness, as indexed by mystical-type experience^4,26^, and longer mindfulness-based meditation periods lead to persisting positive outcomes in healthy subjects^31,60^ and patient populations^4,26,31,61^, we explored whether the intensity of different sub-dimensions of the mystical-type experience predicted persisting changes in behavior and attitudes at a 4-month follow-up. We hypothesized that the combination of psilocybin and mindfulness meditation training would lead to larger changes than meditation alone.

## Results

### State mindfulness and meditation depth

During the 5-day retreat, participants practiced mindfulness meditation, which can be described as a temporary state of intentional self-regulation of attention to foster greater awareness of one’s sensations, emotions, and thoughts with a non-judgmental attitude. On day 4, participants received either a placebo or psilocybin in a double-blind manner.

**Figure 1:**
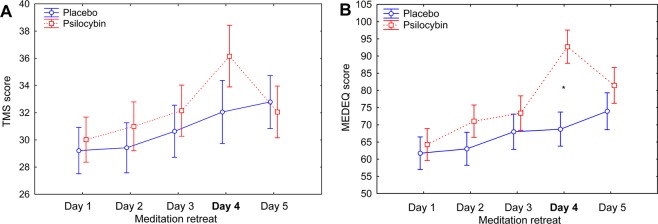
(**A)** Levels of state mindfulness (Toronto Mindfulness Scale total score) and (**B)** meditation depth (Meditation Depth Questionnaire total score) over the course of the retreat. Data are means ± 1 SEM (standard error of the mean). The asterisk indicates a significant difference between psilocybin and placebo groups on day 4, when the drug was administered (*p < 0.01). Both state mindfulness and meditation depth increased between day 1 and day 5 (mindfulness, mean ± SEM: 29.61 ± 1.18 and 32.42 ± 1.36 on day 1 and day 5, respectively, *p* < 0.05; meditation depth, mean ± SEM: 63.0 ± 3.31 and 77.7 ± 3.74 on day 1 and day 5, respectively, *p* < 0.001). The *p*-values have been adjusted for multiple comparisons within this analysis.

Daily, at the end of the meditation session, participants rated the intensity of state mindfulness using the Toronto Mindfulness Scale (TMS). There was no significant interaction between group (placebo/psilocybin) and time (day 1 to 5) for total TMS score (ANOVA *F*(4,148) = 1.05, *p* = 0.38), and no significant main effect of group (*F*(1,148) = 0.41, *p = *0.53), indicating that psilocybin did not significantly increase state mindfulness. However, there was a main effect of time (*F*(4,148) = 4.41, *p* < 0.01. Post-hoc tests showed that total TMS score increased from day 1 to day 5 (mean ± SEM: 29.61 ± 1.18; mean ± SEM: 32.42 ± 1.36; *p* < 0.05; Fig. 1A).

At the end of each meditation session, participants also rated meditation depth using the Meditation Depth Questionnaire (MEDEQ). There was a significant interaction between group (placebo/psilocybin) and time (day 1 to day 5) for MEDEQ score (ANOVA *F*(4,148) = 4.69, *p* < 0.001). Post-hoc tests confirmed the hypothesized increase in mediation depth on day 4 in the psilocybin group was greater than that in the placebo group (*p* < 0.05; Fig. 1). We also found a significant main effect of time (*F*(4,148) = 14.44, *p* < 0.00001), but no main effect of group (*F*(1,37) = 2.44, *p* = 0.13). Post-hoc tests confirmed that MEDEQ score increased from day 1 to day 5 (mean ± SEM: 63.0 ± 3.31 and 77.7 ± 3.74; *p* < 0.001, Fig. 1B).

### Trait mindfulness

**Figure 2:**
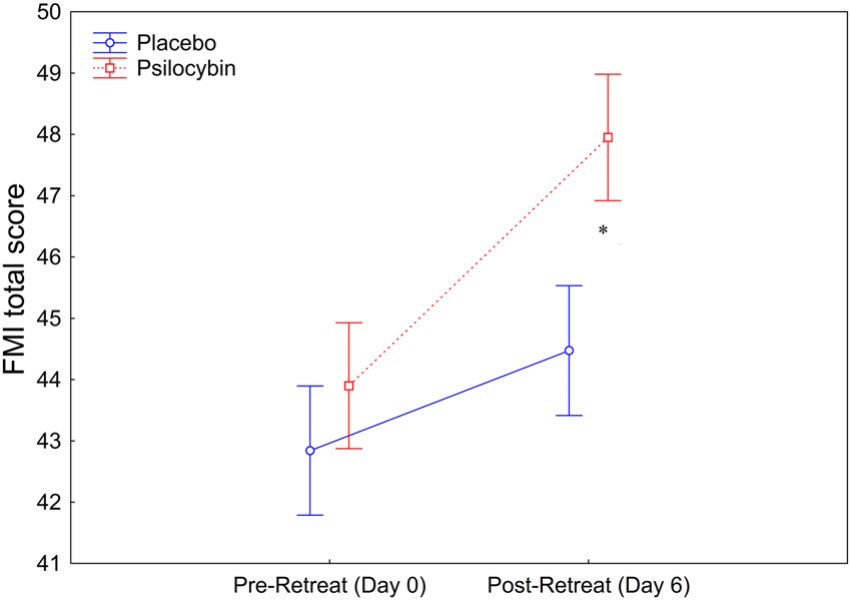
Trait mindfulness (Freiburg Mindfulness Inventory score) pre- and post-retreat. The score was significantly higher in the psilocybin group than in the placebo group on day 6, 2 days after psilocybin administration (*p < 0.001). When collapsed across the two groups (independent of drug treatment), trait mindfulness was significantly higher on day 6 (mean ± SEM: 46.2 ± 0.73) than on day 0 (mean ± SEM: 43.3 ± 0.70). The *p*-values have been adjusted for multiple comparisons within this analysis.

Trait mindfulness was evaluated pre- and post-retreat, on day 0 and day 6, respectively, using the Freiburg Mindfulness Inventory (FMI, short form). There was a significant interaction between group (placebo/psilocybin) and time (pre-retreat, post-retreat) for FMI score (ANOVA *F*(1,37) = 4.14, *p* < 0.05) and a significant main effect of time (*F*(1,37) = 22.85, *p* < 0.0001. Post-hoc tests confirmed that FMI score was higher post-retreat than pre-retreat (*p* < 0.001), and that post-retreat FMI score was higher in the psilocybin group than in the placebo group (*p* < 0.001; Fig. 2).

### Subjective effects during psilocybin-assisted meditation

After psilocybin or placebo administration at 1030 h on day 4, participants engaged in their usual meditation practice, preserving complete silence. The psychological effects of psilocybin peak at about 60–90 min after drug intake, subside gradually, and are absent 6 hours after drug intake^62^. To assess the effect of psilocybin on the subjective experience during the meditation session, participants completed the 5D-ASC and the M-scale. 5D-ASC is designed to quantify positive and negative forms of self/ego-dissolution, including perceptual alterations. The M-scale differentiates between extrovertive self-dissolution (framed by perception of unity and an outward merging with surroundings) and introvertive self-dissolution (framed by ego loss, spacelessness, and/or timelessness, denoting an inward unitary experience beyond time and space)^63,64^.

There was a significant interaction between group (placebo/psilocybin) and 5D-ASC main scales (Oceanic Boundlessness [OB], Visionary Restructuralization [VR], Vigilance Reduction [VIR], Anxious Ego Dissolution [AED], Acoustic Alterations [AA]) for 5D-ASC score (ANOVA *F*(4,148) = 36.27, *p* < 0.00001). Post-hoc tests indicated that the OB (*p* < 0.0001), VR (*p* < 0.0001), and VIR (*p* < 0.05) scores were higher in the psilocybin group than in the placebo group. By contrast, the groups did not differ on the AED and AA scales (SI Table 2).

**Figure 3:**
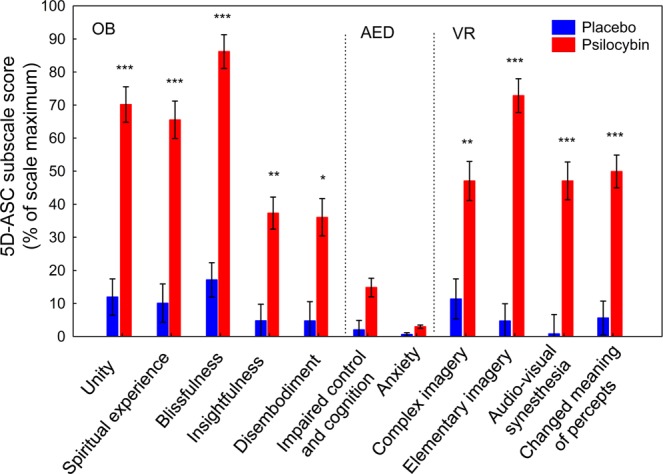
Effects of psilocybin on the subscales of the Altered States of Consciousness Rating Scale (5D-ASC). Scores are expressed as percent of scale maximum. Error bars indicate ± 1 SEM (standard error of the mean). Asterisks indicate significant differences between psilocybin and placebo groups (***p < 0.0001, **p < 0.001, *p < 0.05). OB, Oceanic Boundlessness; AED, Anxious Ego Dissolution; VR, Visual Restructuralization. The *p*-values have been adjusted for multiple comparisons within this analysis.

A subsequent analysis of the subscale scores for the OB, VR, and AED core scales reveled a significant group × subscale interaction (*F*(10,37) = 13.14, *p* < 0.00001). Post-hoc tests indicated that scores for the OB-related subscales of unity, spiritual experience, blissfulness, insightfulness, and disembodiment were higher in the psilocybin group than in the placebo group (all *p* < 0.01). Similarly, scores for the VR-related subscales of complex imagery, elementary imagery, audiovisual synesthesia, and changed meaning of percepts were higher in the psilocybin group than in the placebo group (all *p* < 0.01). By contrast, scores on the AED-related subscales of impaired control and cognition and anxiety did not differ between the two groups (*p* > 0.9; Fig. 3).

**Figure 4:**
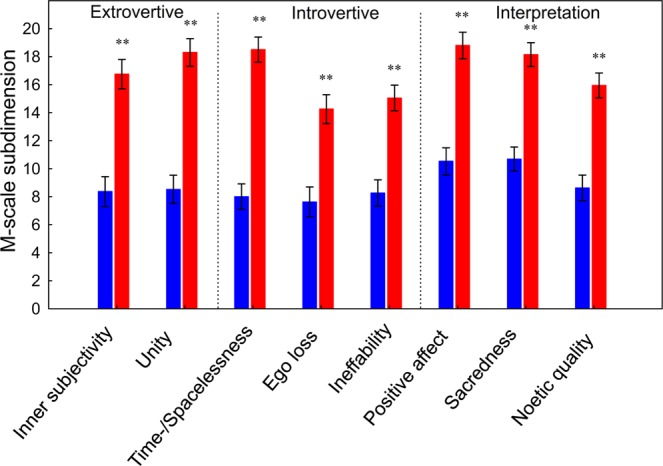
Effect of psilocybin on sub-dimensions of the M-scale. Error bars are ± 1 SEM (standard error of the mean). Asterisks indicate significant differences between psilocybin and placebo groups (**p < 0.001). The *p*-values have been adjusted for multiple comparisons within this analysis.

There was a significant interaction between group (placebo/psilocybin) and dimension (extrovertive mysticism, introvertive mysticism, interpretation) for M-scale score (ANOVA *F*(2,74) = 14.81, *p* < 0.00001). Post-hoc tests indicated that, for all three dimensions, the score was higher in the psilocybin group than in the placebo group (all *p* < 0.01; SI Table 2). Extrovertive mysticism comprises the sub-dimensions unity of the world as one and inner subjectivity of all beings; introvertive mysticism comprises the sub-dimensions of ego loss, timelessness/spacelessness, and ineffability of the experience; and interpretation comprises the sub-dimensions positive affect, sacredness, and noetic quality. Subsequent analysis indicated a significant interaction between group (placebo/psilocybin) and sub-dimension score (F(7,259) = 2.43, *p* < 0.05). Post-hoc tests indicated that, for all sub-dimensions, score was higher in the psilocybin group than in the placebo group (Fig. 4).

**Figure 5:**
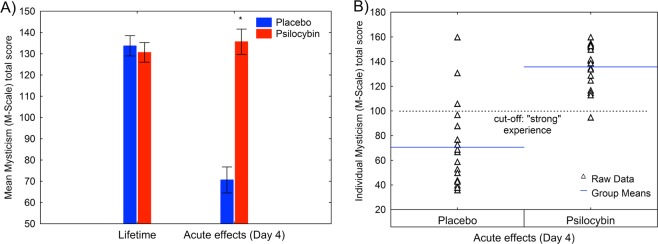
**(A**) M-scale total score for lifetime experience versus acute drug experience for psilocybin and placebo groups. Error bars indicate ± 1 SEM (standard error of the mean). Asterisk indicates a significant difference between psilocybin and placebo groups (*p < 0.01). (**B**) M-scale total score for each participant. The a priori criterion of 60% of scale maximum for a “strong” mystical-type experience was met by 19 of 20 participants (95%) in the psilocybin group and 3 of 19 participants (15.8%) in the placebo group.

**Figure 6:**
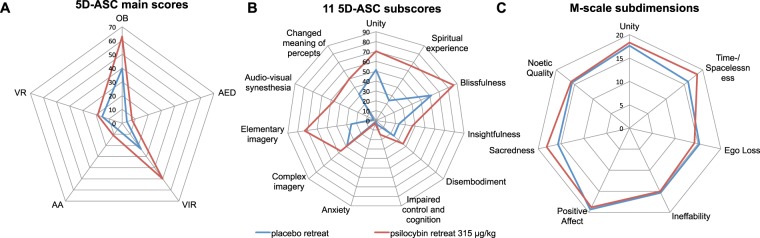
Comparison of subjective effects in participants from the psilocybin group (*n* = 19) with those in the placebo group (*n* = 3), who reached the a priori criteria for a “strong” mystical-type experience. Despite similar M-scale subdimensions (**C**), there were marked differences in various scale and subscale scores of altered states of consciousness (**A**,**B**), particularly in the domain of visual experiences.

Prior to the retreat, participants used the M-scale to rate their strongest lifetime mystical experience. This was then compared with the mystical experience after drug administration during the retreat. There was a significant interaction between group (placebo/psilocybin) and time point (lifetime, retreat) on total M-scale score (ANOVA *F*(1,37) = 68.04, *p* < 0.00001), and a significant main effect of time point (*F*(1,37) = 49.53, *p* < 0.00001) and group (*F*(1,37) = 23.19, *p* < 0.0001; Fig. 5A). Individual M-scale scores revealed that 19 of 20 participants (95%) in the psilocybin group and 3 of 19 participants (16%) in the placebo group met the a priori criteria of 60% of scale maximum for having had a “strong” mystical experience during the retreat (Fig. 5B). Figure 6 depicts in detail the score distribution of altered consciousness and perception for those participants.

The relations between score on each of the main dimensions of the 5D-ASC scale^65^ and each of the three dimensions of the M-scale^66^ were quantified using Pearson’s correlation coefficient. Inspection of the correlation matrix (SI Fig. 2) revealed that score on the OB dimension of the 5D-ASC scale correlated significantly with score on the extrovertive mysticism (*r = *0.47), introvertive mysticism (*r* = 0.59), and interpretation (*r* = 0.59) dimensions of the M-scale (all *p* < 0.01). No other correlations were significant.

### Predictors of acute subjective experience of meditation

Multiple regression analysis was used to explore if dispositional and state variables before drug intake predicted the acute alterations of consciousness (SI Table 4). Specifically, we tested whether age, personality traits (extraversion, neuroticism, and openness to experience), absorption capacity, optimistic attitude toward life, intensity of previous mystical-type experience, trait mindfulness (facets of presence and acceptance), emotion regulation strategies (suppression and reappraisal), state mindfulness, and meditation depth influenced score on the OB, AED, and VR main scales of the 5D-ASC questionnaire and on the extrovertive mysticism, introvertive mysticism, and interpretation dimensions of the M-scale. State mindfulness and meditation depth were assessed the day before drug intake, and all other predictor variables were assessed prior to the start of the retreat. Psilocybin intake accounted for the largest proportion of the variance in all six outcome measures, with *η*2 ranging from 0.35 to 0.74. Optimistic attitude toward life accounted for a significant proportion of the variance in OB, VR, and the three core dimensions of mysticism (*η*^2^ = 0.16–0.25), and openness (*η*^2^ = 0.12–0.18) accounted for a significant proportion of the variance in OB and the three core dimensions of mysticism.

Furthermore, we explored the possibility that emotion regulation strategy and mindfulness practice may prevent anxiety and have a beneficial influence on the psychedelic experience. Indeed, reappraisal of emotions counteracted the intensity of AED (*p* < 0.0001, *η*^2^ = 0.21), and non-judgmental acceptance of thoughts and emotions increased introvertive mysticism (*p* < 0.05 *η*^2^ = 0.14) and interpretation of mystical experience (*p* < 0.05 *η*^2^ = 0.16). Finally, as expected meditation depth accounted for a significant proportion of the variance in OB (*p* < 0.0001, *η*^2^ = 0.38) and interpretation of mystical experience (*p* < 0.05 *η*^2^ = 0.13), notably both dimensions that depict positive emotions associated with self-dissolution.

### Changes in behavior and attitudes at the 4-month follow-up

**Figure 7:**
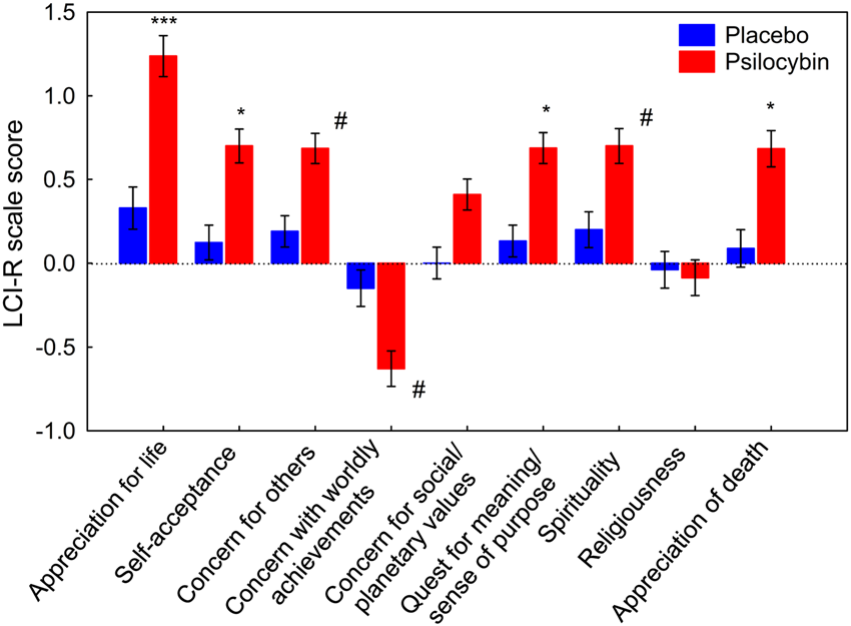
Self-rated changes in attitude and behavior, evaluated using the Life Change Inventory, Revised, at a 4-month follow-up. Error bars indicate ± 1 SEM (standard error of the mean). Asterisks indicate significant differences between psilocybin and placebo groups (^***^p < 0.001, ^*^p < 0.01, ^#^*p* < 0.05–0.07). The *p*-values have been adjusted for multiple comparisons within this analysis.

Four months after the retreat, participants evaluated perceived changes in behavior and attitudes using the Life Changes Inventory, Revised (LCI-R). LCI-R score was significantly higher in the psilocybin group (mean ± SEM: 0.75 ± 0.09) than in the placebo group (mean ± SEM: 0.19 ± 0.05; *F*(1,37) = 23.41, *p* < 0.0001), indicating greater change in attitudes and behavior. There was a significant interaction between group (placebo/psilocybin) and LCI-R scale (*F*(8,296) = 9.28, *p* < 0.00001). Post-hoc tests indicated that scores on the LCI-R scales of appreciation for life, self-acceptance, quest for meaning/sense of purpose, and appreciation of death were significantly higher in the psilocybin group than in the placebo group (all *p* < 0.01). There was also a trend toward a higher score on the concern for others and spirituality scales, and toward a lower score on the concern with worldly achievements scale (e.g., interest in material things, social status) (all *p* < 0.05–0.07; Fig. 7).

Each participant designated a closely related person with whom he/she had frequent contact since the retreat, to complete a third-person LCI-R with reference to the participant. Completed questionnaires were received from 39 data participants and 37 significant others. Two participants declared that there was no closely related person with whom they had frequent contact at that moment of their life. These community observer LCI-R ratings showed a similar, although less marked, overall pattern of changes with significantly higher scores on the self-acceptance and quest for meaning/sense of purpose scales in the psilocybin group than in the placebo group (all *p* < 0.01; SI Fig. 3).

### Meaningfulness of the study experience

At the follow-up, participants were asked how personally meaningful the study experience was. A large proportion of participants in the psilocybin group rated their experience as among either the top ten (50%) or five (35%) most meaningful experiences of their life, whereas the majority of participants in the placebo group rated their experience as meaningful as those experiences occurring once a month (37%), once a year (21%), or every 5 years (26%) (SI Table 3).

### Predictors of changes in attitude and behaviors

A multiple regression was used to test the hypothesis that certain features of the mystical-type experience would predict changes in attitudes and behaviors quantified by the LCI-R at the 4-month follow-up (SI Table 5). Psilocybin intake (*p* < 0.05, *η*^2^ = 0.15) and loss of ego boundaries (*p* < 0.01, *η*^2^ = 0.20) accounted for a significant amount of variance in total LCI-R score. Furthermore, psilocybin intake (*p* < 0.001, *η*^2^ = 0.28) predicted score on the appreciation of death subscale. Sacredness accounted for a significant amount of variance in score on the appreciation of life scale (p < 0.00001, *η*^2^ = 0.48), and sacredness (*p* < 0.05, *η*^2^ = 0.11) together with ineffability (*p* < 0.01, *η*^2^ = 0.17) accounted for a significant amount of variance in score on the quest for meaning/sense of purpose scale. The experience of unity (*p* < 0.001, *η*^2^ = 0.32) accounted for a significant amount of variance in score on the self-acceptance scale.

## Discussion

In the present study, we investigated the synergistic effects of psilocybin and mindfulness as well as related state and trait predictors in experienced meditators during a 5-day group retreat. Given the importance of set and setting in shaping reactions to psychedelics, we hypothesized that mindfulness training may deepen psychedelic-induced experiences. We also investigated the possibility that it may buffer the emotional overload and anxiety that can arise with self-dissolution effects^67^. We showed that state mindfulness and meditation depth gradually increased over the course of the retreat and that meditation depth was acutely increased by psilocybin administration (Fig. 1). These findings demonstrate that during acute drug effects, experienced meditators were able to remain engaged in their usual meditation practice and that psilocybin has the capacity to deepen meditative states. Interestingly, dispositional mindfulness measured 1 day after the completion of the retreat was higher among participants who had received psilocybin than among those who had received placebo (Fig. 2). This finding aligns well with the observed increase in mindfulness capacity 24 hours after administration of the psychedelic N,N-dimethyltryptamine to healthy volunteers in the form ayahuasca^68^. Thus, the current findings corroborate the view that psychedelics may enhance mindfulness capacity^68^ and that dispositional mindfulness may change over time with practice, suggesting putative neuroplasticity-related modulations of this trait^32,69,70^.

The combination of psilocybin and meditation produced much stronger alterations of consciousness and a profound positively experienced self-dissolution (OB score) compared with meditation alone, and virtually without loss of cognitive control and anxiety (AED score), despite the relatively high dose of psilocybin that was used. However, the self-dissolution was accompanied by marked vivid imagery, ranging from elementary to complex scenery hallucinations (VR score). A comparison of the 5D-ASC results in the psilocybin group with those obtained in healthy, non-meditating participants who had conducted a simple neuropsychological task under the same psilocybin dose^44^ revealed that meditation plus psilocybin resulted in substantially higher scores on the spiritual experience (66% vs. 22%), blissful state (86% vs. 48%), and feeling of unity (70% vs. 40%) subscales and lower scores on the AED and VIR scales than psilocybin alone, whereas the auditory and visual alterations did not differ^44^ (SI Fig. 1). These findings support our view that mindfulness-related trait and state capabilities may foster and positively shape the experiential quality of self-dissolution and buffer psilocybin-induced anxiety and vigilance deficits. Interestingly, a 1–2 month preparatory training in meditation and spiritual practice bolstered the positive effects on self-dissolution in healthy individuals, as tested with similar doses of psilocybin^71^.

Three participants in the placebo group met the a priori criteria for a strong mystical-type experience (Fig. 5B). Exploration of M-scale subscales indicated that the mystical-type experience reported by these placebo participants was very similar to that reported by the psilocybin participants. These findings provide the first experimental evidence and corroborate the view^72^ that meditation and psilocybin can occasion phenomenologically overlapping mystical-type experiences. However, there are also some notable differences between meditation- and psilocybin-induced alterations of consciousness. Whereas the mystical-type experiences in the placebo group arose virtually without anxiety, cognitive impairments, audiovisual synesthesia, or vigilance reduction, and with relative few elementary imageries, in the psilocybin group they were associated with vivid visual alterations, pronounced audiovisual synesthesia, moderate cognitive impairments, and vigilance reduction (Fig. 6A,B). Consistent with previous work, such experiences occur in deep meditation unpredictably and at a very low rate^73,74^, whereas psilocybin can occasion such positive mystical-type experiences in a supportive clinical setting at relatively high rates (≈60%)^10,42,44,75^. Psilocybin also produced more intense mystical-type experiences than placebo in the seminal “Good Friday Experiment”, where participants attended a Christian service in a group setting at Harvard University’s Marsh Chapel^10^. In light of these observations, we suggest that the retreat setting in the present study reassured, through the presence of others and social bonding, a safe and supportive environment that may have contributed to the positive outcomes of psilocybin-induced self-dissolution.

Our analysis of putative predictors of the acute psychedelic experience highlights that psilocybin was the most important determinant. This is consistent with a previous pooled analysis of psilocybin experiments^44^. In accordance with earlier studies^46,76,77^, we also found that the personality trait “openness” (expressing the level of permeability to novel experience) and optimism toward life contributed to the experience of OB, as well as the three core dimensions of mysticism. The significant correlation between OB and these three dimensions further supports this finding. Openness is composed of active imagination, attentiveness to inner feelings, intellectual curiosity, preference for variety, adventurousness, and aesthetic sensitivity, clustering together in one dimension^78^. This personality disposition not only predicted the positive outcome of psychedelic experiences^53,60^, but also correlated with the prevalence of spontaneously occurring mystical-type experiences^79^, and was associated with the meditation depth^41^. Consistent with previous work^53^, visionary experience was associated with the personality trait extroversion, but unlike earlier reports it was not correlated with the participant’s absorption capacity^44^. In regard to the core components of mindfulness meditation practice, we found that reappraisal of emotions was negatively related to the AED dimension of altered states of consciousness and introvertive mysticism, which also refers to loss of ego functioning, whereas the mindfulness trait acceptance of thoughts and emotions correlated positively with introvertive mysticism and the interpretation of mystical experience. Furthermore, meditation depth assessed the day before drug administration substantially contributed to the OB dimension of altered states of consciousness and interpretation of mystical experience, both of which depict positive emotions associated with self-dissolution. Relaxation the day before drug administration and age were predictors of positive self-dissolution in a previous study^53^. In light of these observations, it is conceivable that mindfulness training may help to buffer the emotional distress and anxiety that may arise with the ego dissolution state. This may happen by promoting reappraisals, more flexible selection of interpretation of the experience, and redirection of emotional load. A better understanding of non-pharmacological variables could help stratify individuals and better predict specific responses to classical psychedelics.

The follow-up results highlight that changes in behaviors were significantly higher in the psilocybin group than in the placebo group for appreciation of life, self-acceptance, quest for meaning and sense of purpose, and appreciation of death (Fig. 7). The pattern of changes was similar although less pronounced than that obtained from an external observer (SI Fig. 3). This adds to the literature reporting transformational processes following psychedelic experiences. Comparable enduring positive changes in attitude and behavior after one or two doses of psilocybin have been reported to persist at least 14 months^80^ or more^81^. Interestingly, although our participants were well-functioning individuals who scored high in life satisfaction at baseline, it appears that they could still greatly benefit from the psilocybin-facilitated experience.

Remarkably, despite the long engagement of our study participants in contemplative practices, the psilocybin experience was valued equivalent to their strongest lifetime mystical-type experience (Fig. 5A). Moreover, most participants in the psilocybin group attributed a high personal meaning to the psilocybin experience: 37% considered it one of the five, and 47% one of the ten most meaningful life experiences. However, this finding is somewhat less pronounced than that of a recent study in non-meditating psilocybin subjects^42,80^. A possible explanation for this difference is that we administered a lower dose of psilocybin and the long-term meditation practice may support the cultivation of enduring happiness and well-being. Additionally, being open to moment-to-moment experiences, without the aim to judge them and create any attachments, may have contributed to these comparably less pronounced effects on personal meaning.

Our regression analysis of predictors of the long-term behavioral changes showed that the extent of self-dissolution and the drug administration substantially contributed to the global change in attitude and behavior. Moreover, the experience of unity as an index of reduced self-other boundaries and oneness with the surrounding predicted self-acceptance. This temporary loss of the ordinary ego/self and self-boundaries appears to diminish self-referential processing or ego-centricity and thus to foster an altered perspective toward oneself, others, and the environment. This interpretation is supported by recent findings demonstrating that psilocybin modulates neuronal activity in brain networks that mediate a sense of self^8,82^. Furthermore, the sense of sacredness contributed to the change in appreciation of life, and sacredness and ineffability contributed to the change in quest for meaning/sense of purpose, across both groups independent of drug condition. These findings support the view that spiritual experiences, including sense of sacredness and ineffability, contribute to psychological well-being^83–85^, and act through meaning-making mechanisms^86,87^. In line with a recent large survey reporting that peak psychedelic experiences were linked to reduced fear of death^72^, we found that psilocybin alone predicted the change in appreciation of death. This is also consistent with recent studies demonstrating that the extent of psilocybin-occasioned mystical-type experience mediated the reduction of existential anxiety and depression in terminal cancer patients^4,26^.

The perceptible effects of psychoactive drugs pose a methodological challenge in maintaining the integrity of blinding procedures. Therefore, a major limitation of this study was the use of an inert placebo and the possible recognition of the active and non-active conditions by participants. However, this choice was motivated by the objective to quantify the meditation-specific parameters without additional confounding factors, i.e., with respect to the inactive placebo. Nevertheless, we reduced the element of expectancy by including solely psychedelic-naïve volunteers (≈2/3 of the sample) and those with limited previous exposure to consciousness-altering drugs (≈1/3 of the sample). Furthermore, we obscured the experimental conditions (i.e., placebo versus psilocybin) through a highly structured silent retreat that limited interpersonal exchange within the group setting, with instructions emphasizing individual practice. Even though it remains possible that the observed effects reflect some expectancy, we argue based on previous results from our group and other researchers that they are likely attributable to the unique psychoactive profile of psilocybin.

In addition to the overall positive outcome of psilocybin administration found in this study, a few results compel further discussion. At the dose tested, psilocybin produced no adverse events (including physical discomfort, disorientation, severe anxiety, panic, or psychotic-like reactions) neither acutely during the trial session nor post-acutely during the retreat. Two participants reported in the postacute psychiatric investigation that they felt transiently emotionally overwhelmed during the peak effect of the drug, but did not value this as negative. No adverse events or persistent negative effects were reported at the 4-month follow-up. The low ratings on the 5D-ASC scale for “anxiety” (mean score, 3%; min–max range, 0–10%) are in accordance with and even smaller than those reported by previous studies, suggesting that even during peak effects psilocybin is well tolerated and rarely produces profound or psychotic anxiety in a controlled clinical setting in healthy human subjects^6^. However, it should be said that challenging psychological experiences may still occur as acute and durable effects of both meditation practice and psychedelic ingestion, including psychological distress or disorientation^88^, psychotic episodes, panic attacks, depersonalization, or asociality^89^. The incidence of adverse effects can be reduced by proper medical and psychological screening, a reassuring setting, and supervision.

The present study demonstrated that the combination of psilocybin and mindfulness training increased the incidence and intensity of alterations of consciousness characterized by profound states of self-dissolution and virtually no anxiety. Personality traits and core components of mindfulness-based meditation shaped the different facets of selflessness. The psilocybin-induced dissolution of self, either due to a perception of unity of all things and/or ego loss, mediated beneficial enduring changes in psychosocial functioning. The effect of psilocybin on meditation depth and trait mindfulness may increase the effective positive impact of meditation retreats on psychological outcome. Both meditation depth and higher levels of mindfulness have been linked to a wide range of well-being and mental health markers^31,90^. The present results also suggest that the combination of mindfulness training and psychedelic-assisted intervention may offer potential for the further development of psychedelic-assisted applications to improve well-being and health in both therapeutic and non-therapeutic settings

## Methods

### Participants

Participants were 39 expert Buddhist meditation practitioners. The study was advertised as an experiment investigating the effect of psilocybin on mindfulness practice embedded in a 5-day meditation retreat. Participants were recruited through flyers in local meditation communities and advertisements in professional Buddhist magazines devoted to meditation. In total, 202 individuals submitted written applications, of which 79 potentially eligible persons were further screened for criteria for participation over the phone. In the next screening stage, 54 were invited to the clinic for a medical check-up and psychological evaluation, and 40 were finally qualified for the study. Participants were healthy according to medical history, physical examination, routine blood analysis, electrocardiography, and urine tests for drug abuse and pregnancy. The study was approved by the Cantonal Ethics Committee of Zurich, Switzerland. Written informed consent was obtained from every participant before enrollment, and the study was performed according to the Declaration of Helsinki. See Supplemental Information for further information on screening procedures and participation criteria and SI Table 1 for detailed sample characteristics.

### Substance and dosing

The study was conducted using a double-blind, placebo-controlled, between-subject design. Psilocybin (315 μg/kg of body weight; body weight of subjects, 69.07 ± 12.07 kg; absolute dose, 21.76 ± 3.8 mg) and placebo (lactose) were administered in identical gelatin capsules. The high dose of psilocybin was chosen with reference to previous studies, in which a similar dose induced a significant change in consciousness without producing deep thought disturbances or complete loss of self-control^6^. The use of psilocybin according to the study protocol was authorized by the Swiss Federal Office for Public Health, Department of Pharmacology and Narcotics, Bern.

### Study procedures and setting

The 40 participants were randomly allocated to one of two groups (placebo, psilocybin) matched for age, gender, mindfulness level, meditation experience (quantified as lifetime hours of meditation practice), and retreat experience (quantified as number of attended retreats). One participant withdrew after the matching procedure. Two retreats at different times and following the same procedures with 16 (8 placebo/8 psilocybin) and 23 (11 placebo/12 psilocybin) subjects were conducted. During the retreat, the participants engaged in daily, structured meditation sessions in which they sat upright facing a wall, kept their eyes half open, and engaged in meditation practice. On day 4, participants in each group received either placebo or psilocybin, administered in a double-blind manner.

The retreats were held in a meditation center where, for 5 days, the participants lived and practiced meditation under the guidance of an experienced Zen teacher. All individuals followed a structured meditative discipline known in the Zen tradition as *sesshin*. The daily schedule consisted of a 15 hour highly structured program (from 0600 to 2100 h) including typical elements of mindfulness-based Zen practice: 8 × 30 min sitting meditation (*zazen*), 5 × 10 min indoor and 2 × 30 min outdoor walking meditation (*kinhin*), 75 min of mindful physical work (*samu*), interleaved with recitations, short rest breaks, and meals. The participants were instructed to observe silence during the whole retreat. Psilocybin and placebo were administered on the fourth day at 1030 h following a slightly adapted schedule containing additional musical elements and short relaxation periods. Thus, the retreat was conceptually divided into three phases: preparation (days 1–3), psilocybin/placebo administration (proper experience on day 4), and post-experience integration (day 5).

Trait mindfulness was evaluated the day preceding the retreat and the day following the retreat. State mindfulness and meditation depth were evaluated at the end of each day of the retreat (2100 h). Altered states of consciousness and mysticism were evaluated 360 min after drug administration.

Perceived changes in behaviors and attitudes were evaluated at a follow-up, 4 months after the retreat. At this time point, participants also answered the question “*How personally meaningful was the experience made during the study?*” (*1 - no more than everyday experiences; 2 - similar to meaningful experiences that occur on average once or more a week; 3 - once a month; 4 - once a year; 5 - once every 5 years; 6 - among the ten most meaningful experiences of my life; 7 - among the five most meaningful experiences of my life; 8 - the single most meaningful experience of my life*)^9^. Additionally, each participant designated a closely related person with whom he/she had frequent contact since the retreat, such as a family member or close friend, who would likely notice changes in the participant’s behavior and attitudes. This individual was asked to provide a third-person evaluation of changes (if any) in the participant’s behavior and attitudes.

### Baseline measures

At baseline, the following information was collected to characterize and enable matching of the study sample: socio-demographic data, meditation tradition, hours of formal meditation practice, history of drug use, Multiple Choice Vocabulary Test^91^ (verbal fluency intelligence), FMI^92^ long form (dispositional/trait mindfulness, completed with reference to the preceding 2 months), Satisfaction with Life Scale^93^, and Symptom Checklist-90-Revised^94^. The last measure was used as a screening inventory to detect clinical symptoms and indicators of psychological distress and was completed with reference to the last month.

Personality, absorption (propensity to become absorbed in experiences), dispositional optimism and pessimism, emotion regulation strategies, and the occurrence of primary mystical experiences over the course of a lifetime were also evaluated at baseline and considered in the analysis as possible predictors. Personality was evaluated using the NEO Five-Factor Inventory^95^. This is a concise 60-item measure of five basic personality dimensions: extraversion, agreeableness, conscientiousness, neuroticism, and openness to experience. The propensity to become absorbed in experiences was evaluated using the Tellegen Absorption Scale^96^, which consists of 34 items. Dispositional optimism and pessimism were evaluated using the Life Orientation Test, Revised^97^, which consists of ten items. Emotion regulation strategies were evaluated using the Emotion Regulation Questionnaire^98^, which, by means of ten items, evaluates the habitual use of two emotion regulation strategies: cognitive reappraisal and expressive suppression. The occurrence of primary mystical experiences over the course of a lifetime was evaluated using the M-scale (lifetime version)^99^. This 32-item questionnaire derives from Stace’s common criteria of mysticism^100^ and has been extensively used in the field of psychology of religion with well-documented reliability, validity, and cross-cultural generalizability^101,102^. The total score, ranging from 32 (least mystical) to 160 (most mystical), as well as three core dimensions and subdimensions were used for analysis^103^.

### Outcome measures

#### Trait mindfulness

Trait mindfulness was evaluated the day preceding the retreat and the day following the retreat using the FMI short form^104^. The FMI short form is a 14-item reliable instrument assessing the construct of mindfulness closely following Buddhist conceptualization. Mindfulness was treated as a one-dimensional construct, but separate factors of presence and acceptance were also used to refine the predictor analysis. The FMI was completed with reference to the previous 5 days.

#### State mindfulness

State mindfulness was evaluated on each day of the retreat using the TMS^105^, a 13-item measure particularly well-suited to evaluate the level of mindfulness in those who actively practice it. It conceptualizes the construct as an intentional, reflective style of introspection or self-observation. The TMS was completed at the end of the evening meditation session (2100 h) with reference to the meditation practice of the corresponding day.

#### Meditation depth

Meditation depth was evaluated on each day of the retreat using the MEDEQ^39^, which is a 30-item questionnaire designed to capture five aspects of meditation experience: hindrances (e.g., being restless), relaxation (e.g., feeling calm), concentration (e.g., being attentive and detached from emotions and thoughts), transpersonal qualities (e.g., feeling connected, bliss, and grace), and non-dual qualities (e.g., complete rest of thoughts and emotions, experiencing unity of all things). The global MEDEQ score was used for analysis. The instrument was completed at the end of the evening meditation session (2100 h) with reference to the meditation practice of the corresponding day.

#### Altered states of consciousness

Alteration of consciousness was evaluated 360 min after drug administration using the 5D-ASC^65^. This measure comprises 94 visual-analogue items, each rated as a percentage score of a maximum scale value, and was used to quantify the experiential effects of the drug, compared with the placebo. The instrument is a well-validated tool for measuring etiology-independent altered states of consciousness and has been frequently used in previous studies with psilocybin. The global score, the five original scales, and the 11 empirically derived subscales^106^ were quantified. The five original scales are as follows: [OB] Oceanic Boundlessness (derealization and depersonalization, positive affect, altered time perception), [AED] Anxious Ego Dissolution (personal disintegration, loss of self-control, anxiety, thought disturbances), [VR] Visual Restructuralization (visual hallucinations and imagery, synesthesia, changed meaning of percepts), [AA] Auditory Alterations (auditory illusions and hallucinations), and [VIR] Vigilance Reduction (changes in vigilance and alertness). This instrument was completed with reference to drug/placebo experience.

#### Mystical-type experiences

Mystical-type experiences were evaluated around 360 min after drug administration using the M-scale^63^. This comprises three culture-independent core dimensions depicting eight facets of mystical-type experiences: extrovertive mysticism, comprising the sub-dimensions unity of the world as one and inner subjectivity of all beings; introvertive mysticism comprising the sub-dimensions ego loss, timelessness/spacelessness, and ineffability of the experience; and interpretation, comprising the sub-dimensions positive affect, sacredness, and noetic quality. The interpretation dimension reflects a first interpretation of the mystical experience. The scale was found to be sensitive to the effects of psilocybin^9,42^. The following measures were used for analysis: total score, introvertive mysticism score (characterized by an inward-facing ego loss, time and spacelessness, and ineffability), extrovertive mysticism score (relating to the experience of unity of all things and inner subjectivity), interpretation score (referring to a positive mood, sacredness, and noetic quality)^103^, and score on each of the eight original sub-dimensions to allow for comparisons with earlier studies. This instrument was completed with reference to drug/placebo experience.

#### Changes in behavior and attitudes

Changes in behavior and attitudes associated with a possibly transformative event were evaluated at the follow-up time point, 4 months after the retreat, using the LCI-R^107^. The LCI-R was originally used in the context of near-death experiences and adapted for the purpose of this study. The instrument consists of 50 items, each rated on a five-point Likert scale ranging from −2 (strongly decreased) to +2 (strongly increased). The items indicate the change in nine clusters (scales) and combine to create one total change score. Two versions of the instrument were used: a self-report version was completed by participants, and a third-person version was completed by the significant others. Both versions were completed with reference to the 4-month period following the retreat. All questionnaires were anonymized before analysis.

### Statistical analysis

Independent sample *t*-tests and Pearson chi square tests were used to compare continuous and categorical variables, respectively, between the two groups (psilocybin, placebo) to evaluate matching.

A two-way repeated measures ANOVA with group (psilocybin, placebo) as a between-subject factor and dimension or subscale (when appropriate) as a within-subject factor was used to compare the score from the 5D-ASC rating, M-scale, and LCI-R (main scales and subscales of the 5D-ASC, dimensions and subdimensions of the M-scale, and their global/total scores). A two-way repeated measures ANOVA with group (psilocybin, placebo) as a between-subject factor and scale version (lifetime, acute) as a within-subject factor was used to compare the strongest mystical experience across lifespan with the acute psilocybin-induced experience in the study. A two-way repeated measures ANOVA with group (psilocybin, placebo) as a between-subject factor and time (days 1–5) as a within-subject factor was used to assess TMS score and MEDEQ score across the retreat. A two-way repeated measures ANOVA with group (psilocybin, placebo) as a between-subject factor and time (pre-retreat, post-retreat) as a within-subject factor was used to assess FMI score. When significant effects were found, post-hoc pairwise comparisons were performed and *p-* values were adjusted using Tukey’s HSD test.

Pearson’s correlation coefficient was used to quantify the relation between score on each of the main scales of the 5D-ASC and each of the core dimensions of the M-scale, with *p*-values reported after correction for multiple comparisons (SI Fig. 2).

Backward-removal regression analyses were run to evaluate predictors of acute effects (global 5D-ASC score, total M-scale score, score on the OB, AED, and VR scales of the 5D-ASC, and score on the extrovertive mysticism, introvertive mysticism, and interpretation dimensions of the M-scale) and follow-up effects (change in attitudes and behaviors). Regression analyses for acute effects included the following predictors: age, neuroticism, extraversion, openness, absorption, optimism, lifetime mystical experience, presence and acceptance facets of mindfulness, suppression and reappraisal types of emotion regulation, mindfulness as state, and meditation depth on the day of pharmacological intervention, as well as group (placebo/psilocybin). Regression analyses for follow-up effects included the following predictors: score on each of the eight sub-dimensions of the M-scale and group (placebo/psilocybin). The criterion for significance was set at *p* < 0.05.

## Supplementary information

Supplementary Information

## Data Availability

Data is available upon personal request.
