# Supplementary material for: Characterization and prediction of acute and sustained response to psychedelic psilocybin in a mindfulness group retreat
Source: Sci Rep. 2019 Oct 24;9:14914. doi: 10.1038/s41598-019-50612-3 (PMC6813317; doi:10.1038/s41598-019-50612-3)
Supplement: Supplementary file 1 — Supplementary Information [file 41598_2019_50612_MOESM1_ESM.docx]

Supplementary Information for

**Characterization and prediction of acute and sustained response to psychedelic psilocybin in a mindfulness group retreat**

Lukasz Smigielski^1*^, Michael Kometer^1^, Milan Scheidegger^1^, Rainer Krähenmann^1^, Theo Huber^1^, Franz X. Vollenweider^1^

^1^Neuropsychopharmacology and Brain Imaging, Department of Psychiatry, Psychotherapy and Psychosomatics, Psychiatric Hospital, University of Zurich, Lenggstrasse 31, CH-8032 Zurich, Switzerland

^*^Corresponding author: lukasz.smigielski@uzh.ch or lu.smigielski@gmail.com

Content:

Supplementary Text

Tables 1-5

Figures 1-3

References

**Supplementary Information**

**SI Table 1. Study population demographics, screening, and baseline measures**

|  | Placebo group  *n* = 19 | | Psilocybin group  *n* = 20 | | χ^2^ | *p*-value |
| --- | --- | --- | --- | --- | --- | --- |
|  |  | | | | | |
|  |  | |  | |  |  |
|  | N | % | N | % |  |  |
|  |  |  |  |  |  |  |
| Sex |  |  |  |  | 0.041 | 0.84 |
| Male | 11 | 57.9% | 12 | 60.0% |  |  |
| Female | 8 | 42.1% | 8 | 40.0% |  |  |
| Meditation tradition^a^ |  |  |  |  | 0.005 | 0.95 |
| Zen | 16 | 84.2% | 17 | 85.0% |  |  |
| Vipassana | 3 | 15.8% | 3 | 15.0% |  |  |
| Previous drug experience^b^ |  |  |  |  |  |  |
| None | 10 | 52.6% | 11 | 55.0% |  |  |
| ≤3 times, over 20 years ago | 6 | 31.6% | 4 | 20.0% |  |  |
| ≤3 times in the last 20 years | 3 | 15.8% | 5 | 25.0% |  |  |
|  |  |  |  |  |  |  |
|  | M | SE | M | SE | *t* | *p*-value |
|  |  |  |  |  |  |  |
| Age (years) | 50.47 | 2.15 | 52.80 | 1.55 | -0.88 | 0.383 |
| Education (years) | 15.42 | 0.81 | 14.75 | 1.04 | 0.50 | 0.618 |
| Multiple Choice Vocabulary Test score ^c^ | 124.53 | 3.66 | 119.55 | 3.17 | 1.03 | 0.309 |
| Meditation experience |  |  |  |  |  |  |
| Hours of meditation^d^ | 4826.3 | 1339.8 | 6155.0 | 1803.0 | -0.59 | 0.561 |
| Number of retreats^e^ | 30.58 | 11.16 | 31.60 | 6.60 | -0.08 | 0.937 |
| Freiburg Mindfulness Inventory (long form) score | 88.89 | 1.90 | 92.50 | 2.04 | -1.29 | 0.205 |
| Tellegen Absorption Scale score | 68.21 | 5.30 | 75.45 | 3.55 | -1.15 | 0.259 |
| NEO Five Factor Inventory score |  |  |  |  |  |  |
| Neuroticism | 2 | 0.18 | 2.02 | 0.18 | -0.09 | 0.924 |
| Extraversion | 3.22 | 0.17 | 3.07 | 0.17 | 0.63 | 0.527 |
| Openness | 4.3 | 0.11 | 4.25 | 0.13 | 0.31 | 0.761 |
| Life Orientation Test, Revised, score |  |  |  |  |  |  |
| Optimism | 8.37 | 0.43 | 8.70 | 0.54 | -0.47 | 0.638 |
| Meaning in Life Questionnaire score |  |  |  |  |  |  |
| Presence of meaning | 26.53 | 1.26 | 27.05 | 1.31 | 0.29 | 0.775 |
| Search for meaning | 22.95 | 1.78 | 22.00 | 1.95 | -0.36 | 0.722 |
| Emotion Regulation Questionnaire score |  |  |  |  |  |  |
| Suppression | 13.68 | 0.99 | 13.65 | 0.93 | -0.11 | 0.909 |
| Reappraisal | 31.58 | 0.98 | 31.75 | 1.12 | -0.21 | 0.831 |
| Satisfaction with Life Scale score | 25.16 | 0.91 | 26.25 | 1.26 | 0.69 | 0.491 |
| Freiburg Mindfulness Inventory (short form) score |  |  |  |  |  |  |
| Presence | 19.16 | 0.49 | 19.35 | 0.57 | -0.25 | 0.800 |
| Acceptance | 23.68 | 0.63 | 24.55 | 0.71 | -0.91 | 0.367 |
| Lifetime ASC^f^ experience score (M-scale) | 133.74 | 4.67 | 130.65 | 4.71 | 0.47 | 0.645 |
| Symptom Checklist-90-R (Global Severity Index) | 0.33 | 0.04 | 0.27 | 0.05 | -0.91 | 0.371 |

M, mean; SE, standard error. *t*-values are from two-tailed independent sample *t*-tests for continuous variables and Pearson chi square test for categorical variables; *df* = 37.

^a^The only practiced or primary meditation tradition by participants.

^b^Previous experience with consciousness altering drugs (psilocybin, lysergic acid diethylamide (LSD), mescaline, *N*,*N*-dimethyltryptamine (DMT), 3,4-methyl​enedioxy​methamphetamine (MDMA), ketamine, and their derivates; cannabis, and alcohol were not considered).

^c^Measure of verbal fluency intelligence.

^d^Total lifetime hours of formal meditation practice.

^e^Number of meditation retreats lasting at least 3 consecutive days (lifetime).

^f^Altered states of consciousness.

**Participants: screening details**

The health screening included the Symptom Checklist-90-Revised^1^, clinical anamnesis, somatic examination, electrocardiography, and detailed blood analysis. The structured Mini-International Neuropsychiatric Interview^2^ was used to exclude potential axis I and II psychiatric disorders according to the DSM-IV diagnostic guidelines. Exclusion criteria were poor knowledge of German language, major somatic or neurological disorder, present or antecedent psychiatric disorder in the participant or a first-degree relative, alcohol or drug dependence or abuse, current or recent use of medication or drug affecting the nervous system, and pregnancy. Main inclusion criteria were age between 20 and 65 years, no or very limited exposure to hallucinogenic drugs, minimum of 500 hours of lifetime formal meditation practice, Mahayana (Zen) or Theravada (Vipassana) Buddhism as primary meditation background, familiarity with longer periods of meditation in a retreat setting, and for Vipassana practitioners, a previous exposure to Zen-specific elements of meditative discipline. The last criteria ensured that participants would likely comply with the study specific procedures and adapt to physical and emotional rigors of the demanding daily practice. Both Zen and Vipassana practitioners were included due to their resemblance: both are deeply rooted in Buddhist tradition, and both are founded on the cultivation of mindfulness (in Pāli, *sati*)^3^ and have developed a repertoire of similar mental techniques including sitting and walking meditation practices.

To detect a possible recent use of psychoactive substances, a urine test sensitive to amphetamines, benzodiazepines, tetrahydrocannabinol, cocaine, methadone, and opiate metabolites was performed on the first day of the study. For female participants, an additional urine check was conducted to exclude pregnancy. No test was positive for any of these markers. Forty individuals who met the predefined criteria were enrolled in the study. One person withdrew after randomization, and the remaining 39 took part in the experiment. All volunteers were informed about the study procedures and potential risks related to the use of psilocybin. Board and lodging for subjects during the study was free of charge, and study-related travel costs were compensated. No additional monetary incentive was offered for participation.

**Details on randomisation, blinding, and drug administration**

Psilocybin was obtained through the Swiss Federal Office of Health, Department of Narcotics and prepared as gelatin capsules containing 1 and 5 mg of psilocybin by the Hospital Pharmacy of the Canton of Lucerne, which also prepared the respective placebo capsules of identical appearance (containing 1 and 5 mg of mannitol, respectively). The study applied a stratified randomization procedure after all 40 participants had been enrolled in the study. First, subjects were balanced according five strata categories in the following order: age, gender, trait mindfulness level, meditation experience, and retreat experience. Second, subjects were then randomly allocated to the two groups. Third, blinded bottles containing weight-specific dosages (rounded to two decimals places) were prepared by combining 5 and 1 mg psilocybin or placebo capsules, respectively, all of identical appearance. The randomization and blinding were performed by an authorized research physician in collaboration with the University Hospital Pharmacy who had no further role in the study. All collected raw data were anonymized and transferred to our licensed Clinical Trial Unit, where they were coded into a GCP-based electronic data base protected by double-login control by a research assistant who had no further role in the subsequent statistical data analysis. Data transfer and data quality were guaranteed in a second step by the clinical study manager. For the subsequent statistical analysis, data were initially coded as group 1 and 2, and finally un-blinded for interpretation of the results.

**SI Table 2. Subjective effects measured using the Altered States of Consciousness Rating Scale (5D-ASC) and Mysticism Scale (M-scale)**

| Questionnaire | Scale / dimension | Placebo | | Psilocybin | |
| --- | --- | --- | --- | --- | --- |
|  |  | Mean (SE) | Range | Mean (SE) | Range |
| **5D-ASC** |  |  |  |  |  |
|  | Oceanic Boundlessness | 10.94 (3.44) | 0–47.81 | 62.95 (4.82)*** | 10.41–97.04 |
|  | Visionary Restructuralization | 5.40 (2.21) | 0–31.78 | 49.96 (4.61)*** | 5.94–81.67 |
|  | Anxious Ego Dissolution | 1.74 (0.53) | 0–9.53 | 7.59 (1.36) | 0.95–24.90 |
|  | Auditory Alterations | 1.65 (0.59) | 0–9.44 | 10.88 (2.09) | 0.88–33.44 |
|  | Vigilance Reduction | 4.24 (1.38) | 0–19.83 | 18.57 (2.77)* | 6.42–52.75 |
| **M-scale** | Extrovertive | 25.37 (3.38) | 12–60 | 52.65 (1.78)** | 32–60 |
|  | Introvertive | 15.42 (1.84) | 8–40 | 30.10 (1.53)** | 12–40 |
|  | Interpretation | 29.84 (2.89) | 16–60 | 52.90 (1.39)** | 40–60 |

SE, standard error. Asterisks indicate significant differences between psilocybin and placebo groups (*** = *p* < 0.0001, ** = p < 0.001, * = *p* < 0.05). The *p*-values have been adjusted for multiple comparisons within this analysis.

**Explorative comparison of setting effects**

To further explore the putative synergistic effects of psilocybin and meditation, the score on each of the 11 5D-ASC subscales was compared between the psilocybin group in the present study (*n* = 20) and participants from a previous study on the effect of psilocybin in non-meditators who completed four simple neuropsychological tasks in a laboratory setting (*n* = 20)^4^. An explorative analysis of covariance with group (psilocybin retreat/psilocybin laboratory) as a between-subjects factor, 5D-ASC subscale score as a within-subject factor, and age as a covariate revealed a significant group × subscale interaction (*F*(10,350) = 2.09, *p <* 0.05) with no main effect of age (*F*(35,1) = 0.42, *p* = 0.52). Post-hoc tests indicated that the scores for the unity, spiritual experiences, and blissfulness were higher for the psilocybin retreat group than for the psilocybin laboratory group, while the score for the audio-visual synesthesia was lower for the first group than for the second group (SI Fig. 1).

**SI Figure 1.** Radar chart comparing the score for each subscale of altered states of consciousness in the psilocybin retreat group (*n* = 20) and non-meditators who took the same dose of psilocybin (*n* = 20) in laboratory setting from our database^4^ (* = *p* < 0.05, ** = *p* < 0.01, *** *p* < 0.001). For further comparison, the scores of the three placebo subjects who met the a priori criteria for a strong mystical-type experience are also shown. The *p*-values have been adjusted for multiple comparisons within this analysis.

**SI Figure 2.** Correlations between scores on each dimension of the Altered Stated of Consciousness Rating Scale (5D-ASC) and M-scale. Score on the Oceanic Boundlessness (OB) scale of the 5D-ASC correlated significantly with the score on the extrovertive mysticism (*r* = 0.47), introvertive mysticism (*r* = 0.59), and interpretation (*r* = 0.59) dimensions of the M-scale (all *p* < 0.01). No other correlations were significant. VR, Visual Restructuralization; AED, Anxious Ego Dissolution; AA, Auditory Alterations; VIR, Vigilance Reduction.

**SI Figure 3.** Changes in attitude and behavior, evaluated by a significant other using the Life Changes Inventory, Revised, at a 4-month follow-up. Bars are means and error bars indicate SEM (standard error of the mean). Asterisks indicate significant differences between psilocybin and placebo groups: * = *p* < 0.01. The *p*-values have been adjusted for multiple comparisons within this analysis.

**SI Table 3. Meaningfulness of study experience**

| ***How personally meaningful was the***  ***experience made during the study?*** | Placebo group Psilocybin group |
| --- | --- |
|  | *n* = 19 % *n* = 20 % |
| *1. No more than everyday experiences*  1 5.3% 0 0%  *2. Similar to meaningful experiences that occur on average once or more a week* 1 5.3% 0 0%  *3. Similar to meaningful experiences that occur on average once or more a month* 7 36.8% 0 0%  *4. Similar to meaningful experiences that occur on average once a year* 4 21.1% 1 5%  *5. Similar to meaningful experiences that occur on average once every 5 years*  5 26.3% 2 10%  *6. Among the ten most meaningful experiences of my life* 1 5.3% 10 50%  *7. Among the five most meaningful experiences of my life* 0 0% 7 35%  *8. The single most meaningful experience of my life* 0 0% 0 0% | |

*Note:* percentage (*%*) and number (*n*) of participants rating the question on personal meaning of the overall retreat experience.

**SI Table 4. Predictors of psychological responses evaluated using main scales of the 5D-ASC and main dimensions of the M-scale**

**Oceanic Boundlessness Anxious Ego Dissolution Visual Restructuralization Extrovertive Mysticism Introvertive Mysticism Interpretation**

|  | | | | | | | | | | | | | | | | | | | | | | | | | | | | | | |
| --- | --- | --- | --- | --- | --- | --- | --- | --- | --- | --- | --- | --- | --- | --- | --- | --- | --- | --- | --- | --- | --- | --- | --- | --- | --- | --- | --- | --- | --- | --- |
| **Whole model** | *R^2^adj* | *df* | *F* | *p <* |  | *R^2^adj* | *df* | *F* | *p <* |  | *R^2^adj* | *df* | *F* | *p <* |  | *R^2^adj* | *df* | *F* | *p <* |  | *R^2^adj* | *df* | *F* | *p <* |  | *R^2^adj* | *df* | *F* | *p <* |  |
|  | 0.82 | 5,33 | 36.54 | 0.00001 |  | 0.41 | 2,36 | 14.27 | 0.0001 |  | 0.75 | 3,35 | 39.35 | 0.00001 |  | 0.69 | 4,34 | 22.63 | 0.00001 |  | 0.65 | 6,32 | 12.92 | 0.00001 |  | 0.80 | 8,30 | 20.16 | 0.00001 |  |
| **Multiple Regression**  *backward removal* |  |  |  |  |  |  |  |  |  |  |  |  |  |  |  |  |  |  |  |  |  |  |  |  |  |  |  |  |  |  |
|  | *β* | *df* | *F* | *p <* | *η^2^* | *β* | *df* | *F* | *p <* | *η^2^* | *β* | *df* | *F* | *p <* | *η^2^* | *β* | *df* | *F* | *p <* | *η^2^* | *β* | *df* | *F* | *p <* | *η^2^* | *β* | *df* | *F* | *P <* | *η^2^* |
| **Predictor** |  |  |  |  |  |  |  |  |  |  |  |  |  |  |  |  |  |  |  |  |  |  |  |  |  |  |  |  |  |  |
| Age |  |  |  |  |  |  |  |  |  |  |  |  |  |  |  |  |  |  |  |  |  |  |  |  |  | 0.26 | 1 | 10.76 | 0.01 | 0.26 |
| Neuroticism |  |  |  |  |  |  |  |  |  |  |  |  |  |  |  |  |  |  |  |  |  |  |  |  |  |  |  |  |  |  |
| Extraversion |  |  |  |  |  |  |  |  |  |  | 0.25 | 1 | 9.13 | 0.01 | 0.21 |  |  |  |  |  |  |  |  |  |  |  |  |  |  |  |
| Openness | 0.19 | 1 | 7.12 | 0.01 | 0.18 |  |  |  |  |  |  |  |  |  |  | 0.21 | 1 | 4.84 | 0.05 | 0.12 | 0.22 | 1 | 4.82 | 0.05 | 0.13 | 0.20 | 1 | 6.62 | 0.05 | 0.18 |
| Absorption | 0.18 | 1 | 4.41 | 0.05 | 0.12 |  |  |  |  |  |  |  |  |  |  |  |  |  |  |  |  |  |  |  |  |  |  |  |  |  |
| Optimism | 0.24 | 1 | 10.71 | 0.01 | 0.25 |  |  |  |  |  | 0.27 | 1 | 10.27 | 0.01 | 0.23 | 0.24 | 1 | 6.65 | 0.01 | 0.16 | 0.27 | 1 | 7.25 | 0.01 | 0.18 | 0.26 | 1 | 11.17 | 0.01 | 0.27 |
| Lifetime mysticism |  |  |  |  |  |  |  |  |  |  |  |  |  |  |  | 0.31 | 1 | 11.56 | 0.01 | 0.25 | 0.29 | 1 | 7.84 | 0.01 | 0.20 | 0.22 | 1 | 5.94 | 0.05 | 0.17 |
| Mindfulness: Presence |  |  |  |  |  |  |  |  |  |  |  |  |  |  |  |  |  |  |  |  |  |  |  |  |  | -0.19 | 1 | 4.19 | 0.05 | 0.12 |
| Mindfulness: Acceptance |  |  |  |  |  |  |  |  |  |  |  |  |  |  |  |  |  |  |  |  | 0.27 | 1 | 5.00 | 0.05 | 0.14 | 0.22 | 1 | 5.85 | 0.05 | 0.16 |
| Emotion Regulation: S |  |  |  |  |  |  |  |  |  |  |  |  |  |  |  |  |  |  |  |  |  |  |  |  |  |  |  |  |  |  |
| Emotion Regulation: R |  |  |  |  |  | -0.39 | 1 | 9.58 | 0.01 | 0.21 |  |  |  |  |  |  |  |  |  |  | -0.30 | 1 | 6.29 | 0.05 | 0.16 |  |  |  |  |  |
| State Mindfulness* |  |  |  |  |  |  |  |  |  |  |  |  |  |  |  |  |  |  |  |  |  |  |  |  |  |  |  |  |  |  |
| Meditation Depth* | 0.42 | 1 | 20.27 | 0.0001 | 0.38 |  |  |  |  |  |  |  |  |  |  |  |  |  |  |  |  |  |  |  |  | 0.21 | 1 | 4.58 | 0.05 | 0.13 |
| Group placebo-psilocybin | 0.68 | 1 | 84.18 | 0.00001 | 0.72 | 0.55 | 1 | 19.46 | 0.0001 | 0.35 | 0.81 | 1 | 98.73 | 0.00001 | 0.74 | 0.78 | 1 | 74.74 | 0.00001 | 0.69 | 0.69 | 1 | 48.76 | 0.00001 | 0.60 | 0.63 | 1 | 55.27 | 0.00001 | 0.65 |

*N Note: df,* degrees of freedom*; R^2^ajd*, *R^2^* adjusted; *η^2^*, partial eta squared; *, drug session; R, Reappraisal; S, Suppression.

**SI Table 5. M-scale dimensions as predictors of behavioral changes evaluated using the Life Changes Inventory, Revised**

**LCI-R total score LCI-R Appreciation for life LCI-R Self-acceptance LCI-R Quest for meaning Appreciation of death**

| **Whole model** | *R^2^adj* | *df* | *F* | *p <* |  | *R^2^adj* | *df* | *F* | *p <* |  | *R^2^adj* | *df* | *F* | *p <* |  | *R^2^adj* | *df* | *F* | *p <* |  | *R^2^adj* | *df* | *F* | *p <* |  |
| --- | --- | --- | --- | --- | --- | --- | --- | --- | --- | --- | --- | --- | --- | --- | --- | --- | --- | --- | --- | --- | --- | --- | --- | --- | --- |
|  | 0.48 | 2,36 | 18.70 | 0.00001 |  | 0.47 | 1,37 | 34.45 | 0.00001 |  | 0.30 | 1,37 | 17.64 | 0.001 |  | 0.49 | 2,36 | 19.58 | 0.00001 |  | 0.26 | 1,37 | 14.64 | 0.001 |  |
| **Multiple Regression** *backward removal* |  |  |  |  |  |  |  |  |  |  |  |  |  |  |  |  |  |  |  |  |  |  |  |  |  |
|  | *β* | *df* | *F* | *p <* | *η^2^* | *β* | *df* | *F* | *p <* | *η^2^* | *β* | *df* | *F* | *p <* | *η^2^* | *β* | *df* | *F* | *p <* | *η^2^* | *β* | *df* | *F* | *p <* | *η^2^* |
| **Predictors**  **(M-Scale dimensions)** |  |  |  |  |  |  |  |  |  |  |  |  |  |  |  |  |  |  |  |  |  |  |  |  |  |
| Inner subjectivity |  |  |  |  |  |  |  |  |  |  |  |  |  |  |  |  |  |  |  |  |  |  |  |  |  |
| Unity |  |  |  |  |  |  |  |  |  |  | 0.57 | 1 | 17.64 | 0.001 | 0.32 |  |  |  |  |  |  |  |  |  |  |
| Time/Spacelessness |  |  |  |  |  |  |  |  |  |  |  |  |  |  |  |  |  |  |  |  |  |  |  |  |  |
| Ego loss | 0.43 | 1 | 8.97 | 0.01 | 0.20 |  |  |  |  |  |  |  |  |  |  |  |  |  |  |  |  |  |  |  |  |
| Ineffability |  |  |  |  |  |  |  |  |  |  |  |  |  |  |  | 0.44 | 1 | 7.48 | 0.01 | 0.17 |  |  |  |  |  |
| Positive affect |  |  |  |  |  |  |  |  |  |  |  |  |  |  |  |  |  |  |  |  |  |  |  |  |  |
| Sacredness |  |  |  |  |  | 0.69 | 1 | 34.45 | 0.00001 | 0.48 |  |  |  |  |  | 0.34 | 1 | 4.49 | 0.05 | 0.11 |  |  |  |  |  |
| Noetic quality |  |  |  |  |  |  |  |  |  |  |  |  |  |  |  |  |  |  |  |  |  |  |  |  |  |
| Group placebo-psilocybin | 0.37 | 1 | 6.44 | 0.05 | 0.15 |  |  |  |  |  |  |  |  |  |  |  |  |  |  |  | 0.53 | 1 | 14.64 | 0.001 | 0.28 |

*Note: df,* degree of freedom; *R^2^ajd*, *R^2^* adjusted; *η^2^*, partial eta squared.

**References:**

1 Derogatis, L. *SCL-90-R: Symptom Checklist-90-R: administration, scoring, and procedures manual*. (NCS Pearson, 1996).

2 Sheehan, D. V. *et al.* The Mini-International Neuropsychiatric Interview (M.I.N.I.): the development and validation of a structured diagnostic psychiatric interview for DSM-IV and ICD-10. *Journal of Clinical Psychiatry* **59 Suppl 20**, 22-33 (1998).

3 Chiesa, A. & Malinowski, P. Mindfulness-based approaches: are they all the same? *J Clin Psychol* **67**, 404-424, doi:10.1002/jclp.20776 (2011).

4 Studerus, E., Kometer, M., Hasler, F. & Vollenweider, F. X. Acute, subacute and long-term subjective effects of psilocybin in healthy humans: a pooled analysis of experimental studies. *Journal of Psychopharmacology* **25**, 1434-1452 (2011).
